# Spontaneous Pneumothorax in a Patient with Systemic Lupus Erythematosus and Recent Infection with Coronavirus

**DOI:** 10.1155/2022/9594063

**Published:** 2022-08-22

**Authors:** Nicholas Graves, Julia Flint, Amol Sagdeo, Ayman Askari, Patrick Ball, Hana Morrissey

**Affiliations:** ^1^School of Medicine, David Weatherall Building, University Road, Keele University, Staffordshire ST5 5BG, UK; ^2^The Robert Jones and Agnes Hunt Orthopaedic Hospital, NHS Foundation Trust, SY10 7AG, UK; ^3^The University of Wolverhampton, Faculty of Science and Engineering, School of Pharmacy, Wulfruna Street, WV1 1LY, UK

## Abstract

A 50-year-old woman with a history of systemic lupus erythematosus and a recent infection with COVID-19 presented to the emergency department with acute shortness of breath twice in 10 days. She was diagnosed with myopericarditis attributed to COVID-19 infection (first admission), and chest X-ray revealed a small left-sided pneumothorax, pericardial effusion (second admission), with no mediastinal shift or other signs of tension. Computed tomography confirmed these results and revealed a few small cysts in the right lung. An echocardiogram demonstrated normal heart anatomy and filling dynamics. The patient was diagnosed with simple pneumothorax and ongoing myopericarditis managed with colchicine, ibuprofen, and low-dose prednisolone. The patient responded to treatment and was discharged. Pneumothorax association with COVID-19 is reported in a small number of publications, but the association is less clear with SLE. Our patient may have been predisposed to developing pneumothorax after COVID-19 infection due to her existing connective tissue disorder.

## 1. Background

Pneumothorax occurs due to air accumulation in the thoracic cavity. Significant accumulations may increase pressure and cause lung collapse [1]. Pneumothoraces may occur spontaneously or due to trauma. They are further divided into simple or tension pneumothorax. The latter is a medical emergency, as displacement of the trachea and mediastinal structures may compromise cardiac and pulmonary function [2]. Spontaneous pneumothorax is associated with a variety of etiologies including chronic lung disease, such as chronic obstructive pulmonary disease, and cigarette smoking [1]. More recently, an association has been identified between COVID-19 and pneumothorax with a number of published case reports and series [1, 2]. It is less clear whether systemic lupus erythematosus (SLE) can predispose to pneumothorax, with relatively limited literature linking the two. There is, however, a theoretical potential relationship between any connective tissue disorder and a reduction of the integrity of the pleural membranes resulting in pneumothorax [3, 4]. SLE is characterized by the production of antibodies directed against self-antigens, especially double-stranded DNA (dsDNA) [3]. The target organ damage arises primarily due to inflammatory response to immune complex deposition. The deposition of these immune complexes activates the immune system via complement activation and Fc-receptor binding. This results in the production of inflammatory cytokines, the recruitment of immune cells such as macrophages, as well as elevation in the erythrocyte sedimentation rate (ESR) [4]. While pneumothorax has rarely related to SLE, inflammation of the pleura is a recognized complication [5]. Mechanistically, immune-complex induced inflammatory damage to the visceral pleura could allow the entry of air into the pleural space and consequent pneumothorax. We present a case of a woman with existing drug-induced lupus and recent infection with COVID-19 who developed a spontaneous pneumothorax and myopericarditis [5].

## 2. Ethics

This case report received a clearance from the NHS Trust ethics committee on 22/4/2022.

## 3. Case Presentation

A 50-year-old woman (XY) presented to the emergency department (ED) at a national health service (NHS) hospital trust in England, following several days of shortness of breath, which had suddenly worsened over the previous hour with the patient having increased palpitations. The patient was admitted as she was dyspneic, using accessory muscles of respiration, respiratory rate was 17 breaths per minute, and oxygen saturation was 99% in air. The trachea was central, but on auscultation, breath sounds were absent in the left upper zone. Her body temperature was not raised. She was hemodynamically stable; her blood pressure was 115/74 mmHg, and her pulse rate was 56 bpm. Of note, the patient was double vaccinated against COVID-19 and had also received a booster. XY had been discharged from another NHS hospital trust 10 days prior, after a similar episode of chest pain and dyspnea, diagnosed as myopericarditis with pericardial effusion. This was attributed to a recent COVID-19 infection, three months before her first admission. The past medical history was significant for complex post-traumatic stress disorder, mixed depression and anxiety, drug-induced lupus erythematosus, and recent calculus cholecystitis.

## 4. Investigations

**Figure 1:**
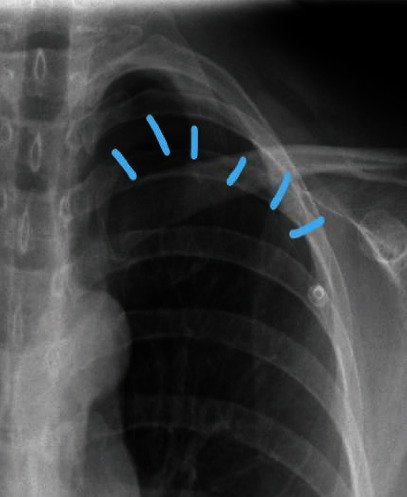
Chest X-ray.

An initial chest radiograph demonstrated a left upper zone pneumothorax, which occupied about 20% of the volume of the left hemithorax. There was no mediastinal shift or signs of tension. There were however some consolidative changes at the base of the left lung, believed to be related to the preceding COVID-19 infection (Figure 1).

**Figure 2:**
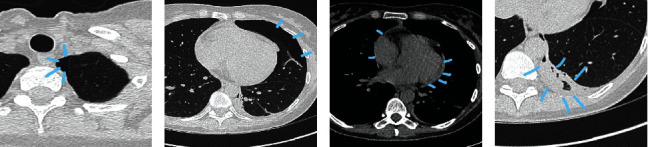
CT of patient XY chest ((a), (b) pneumothorax, (c) pericardial effusion, and (d) left lower lobe consolidation).

The full blood count was largely unremarkable, no neutrophilia, and normal hemoglobin and platelets. Electrolytes were normal as were the liver function tests (GGT of 58 *μ*mol/L). Coagulation screen was also normal. Troponin T was slightly raised at 26 ng/L; C-reactive protein level was measured on 3rd day of admission and was marginally raised at 6 mg/L. Measurements for Complement components 3 and 4 (C3/C4), anti-dsDNA and ESR were within normal ranges. The patient was negative for HIV, hepatitis B/C, and TB. A computed tomography (CT) thorax confirmed the X-ray findings of pneumothorax and consolidation (Figures 2(a) and 2(b)). There were a few small cysts in the right upper and lower lobes but no generalized bullae. A small pericardial effusion was seen (Figures 2(c) and 2(d)). There was no evidence of cardiomegaly.

An ECG revealed normal sinus rhythm with frequent ectopic beats. An echocardiogram demonstrated normal heart anatomy and filling dynamics. XY displayed the unique butterfly rash associated with SLE.

## 5. Management

As above, management was based upon a diagnosis of pneumothorax, myopericarditis with effusion, and lung consolidation; the latter is likely reflecting pneumonia related to COVID-19. Due to the size and lack of any signs of tension, the pneumothorax was managed conservatively, as the risks of needle aspiration were considered to outweigh the potential benefits. This was in line with the British Thoracic Society guidelines for the management of pneumothorax [6]. A course of doxycycline 200 mg stat and 100 mg twice daily for 10 days was commenced to treat the infective consolidation. Colchicine 500 micrograms once daily and ibuprofen 400 mg three times a day were commenced to treat the myopericarditis [7]. Her lupus medications, prednisolone 5 mg once daily and hydroxychloroquine alternating between 200 mg and 400 mg once daily, were continued. Her normal psychiatric medications, quetiapine 300 mg once daily, venlafaxine XL 225 mg once daily, and diazepam 5 mg up to twice daily as needed, were also continued throughout her hospital admission.

## 6. Discussion

Spontaneous pneumothorax has emerged as a relatively uncommon complication of infection with COVID-19. It has rarely been associated with SLE. In fact, a PubMed search of the terms “pneumothorax” and “systemic lupus erythematosus” returned only 27 results. However, pleuritis has been more frequently documented in SLE with risk factors including high levels of anti-dsDNA and lower respiratory tract infection [8]. The underlying mechanism of how SLE may contribute to the development of pneumothorax is unclear.

Out of a total of 27 search results returned, 12 were excluded due to lack of relevance, and 3 others were excluded as they were unpublished conference proceedings, leaving 12 case reports over the past 47 years. The results are summarized in Table 1.

From these results, there have been several reports of pneumothorax associated with SLE. It remains a rare complication of the disease, but it is possible that the underlying rheumatologic disease predisposed the patient to develop pneumothorax when challenged by a severe viral infection such as COVID-19.

The patient also developed pericarditis following infection with COVID-19. A number of reports have been published that appear to show an association between the two conditions. A PubMed search using the terms “COVID-19” and “pericarditis” yielded 233 results. A search using the terms “COVID-19” and “pericarditis” and “pericardial effusion” yielded 47 results (both searches were conducted on 21/3/2022). It is likely that inflammation of the pericardium is a recognized complication of infection with COVID-19.

An important issue that arose was how to manage psychosis in the context of acute myopericarditis on a background of SLE, which would be difficult without the option of corticosteroids. Glucocorticoids may induce psychosis, especially in vulnerable patients [21]. Our patient, XY, had a clear history of psychosis, which obviously had the potential to complicate management. Therefore, the therapeutic strategy was to mitigate the risk of psychosis, by any or all of the following: administration of antipsychotics prophylactically, the use of steroid-sparing agents to reduce the steroid dose needed, and timing the dosing to reduce the risk of psychosis. XY was already taking quetiapine, venlafaxine, and diazepam which were continued throughout her hospital admission. This may have reduced the risk of a psychotic relapse. Adjunctive medications, for example, benzodiazepines, may also be needed if their psychosis presented with severe aggression or agitation [22]. XY was also only treated with a small prednisolone dose of 5 mg, taken in the morning. This, along with her concurrent use of antipsychotic medication, proved successful.

## 7. Conclusion

This case illustrates complex presentation and management issues.

There are no formal management guidelines that fully address this complex setting, although the BTS Guidelines for the management of pneumothorax [6] appear to have been appropriate to this case. Good clinical judgment may reduce the risk of psychosis in such complex patients. Maintaining her other medications is believed to have helped to prevent a psychotic relapse. Furthermore, use of a small dose of prednisolone taken in the morning probably also supported the risk reduction.

Optimizing the underlying connective tissue disease in patients when they present with respiratory complications is important. It should also be noted that these patients may be at greater risk of structural pulmonary complications, such as pneumothorax, when they develop acute respiratory infection. Awareness of this may assist clinicians.

## Figures and Tables

**Table 1:** Summary of case reports identified.

Reference	Demographics	Outcome
Richards et al., 1975 [9]	34 years, female	Death (cardiac arrest)
Passero and Myers, 1980 [10]	27 years, male	Death
Masuda et al., 1990 [11]	41 years, female	Death
Paira et al. 1992 [12]	36 years, male	Death
Nishitsuji et al., 1998 [13]	23 years, male	Survived
Yen et al., 2001 [14]	17, female	Survived
Wilhelm et al., 2002 [15]	17, male	Survived
Maeda et al., 2009 [16]	53, female	Survived, no recurrence
Tanaka et al., 2010 [17]	37, female	Survived
Dalili et al., 2014 [18]	19, female	Death (respiratory failure)
Pamuk et al., 2014 [19]	21, female	Survived
Jatwani et al., 2018 [20]	81, male	Survived

## Data Availability

No other data was used in this case; all data is reported in the manuscript.
